# Surface enhanced Raman spectroscopy as a novel tool for rapid quantification of heroin and metabolites in saliva

**DOI:** 10.3906/sag-1912-196

**Published:** 2020-08-26

**Authors:** Ramazan AKÇAN, Mahmut Şerif YILDIRIM, Hasan İLHAN, Burcu GÜVEN, Uğur TAMER, Necdet SAĞLAM

**Affiliations:** 1 Department of Forensic Medicine, Faculty of Medicine, Hacettepe University, Ankara Turkey; 2 Department of Forensic Medicine, Faculty of Medicine, Afyonkarahisar Health Sciences University, Afyonkarahisar Turkey; 3 Department of Nanotechnology and Nanomedicine, Graduate School of Science and Engineering, Hacettepe University, Ankara Turkey; 4 Department of Food Engineering, Faculty of Engineering, Hacettepe University, Ankara Turkey; 5 Department of Analytical Chemistry, Faculty of Pharmacy, Gazi University, Ankara Turkey

**Keywords:** Heroin and morphine identification, Heroin and morphine quantification, surface-enhanced Raman spectroscopy (SERS), Raman-active surface, gold nanorod arrays, silver nanoparticles

## Abstract

**Background:**

Heroin can be detected and quantified by certain analytical methods, however, forensic professionals and criminal laboratories study for cheaper and faster detection tools. Surface-enhanced Raman spectroscopy (SERS) rises as a possible alternative tool with its widening application spectra. There are few studies regarding Raman and SERS spectra of heroin and its metabolites, which are unfortunately controversial. In this study, we compared five different surfaces in order to find out more efficient Raman-active substrate for opiate detection and rapid quantification of heroin and its metabolites in saliva.

**Materials and methods:**

Morphine standard material was used to identify proper surface for SERS analysis of opiates. Heroin and its metabolites (morphine, morphine-3-ß-glucuronide and 6-monoacetyl morphine) were calibrated between 50 ppb and 500 ppm and quantified on AuNRs with signal enhancement of silver colloids in saliva. Raman microscope with a 785-nm laser source was used.

**Results and Conclusion:**

Obtained results showed that heroin and its metabolites can be detected and quantified in saliva samples using a SERS-based system. Additionally, the present study revealed that synergetic effect of a specific gold nano-surface with ability controlling liquid motion and silver nanoparticles increase band numbers and intensities. Therefore, we suggest a fast, accurate and cost-effective method to detect and quantify heroin in biological fluids.

## 1. Introduction

Abuse of illicit drugs, including high-risk opioids, is still a major problem globally. It is responsible from increasing mortality and morbidity, particularly in young population. Heroin is one of the most commonly abused illicit drugs, which is diacetyl product of morphine that most commonly extracted from *Papaver somniferum* [1]. In a survey study from the United States [2], it has been stated that heroin use is extending in the population from city centers to urban areas and to different socio-cultural classes. According to World Drug Report 20171United Nations Office on Drugs and Crime (2018). World Drug Report 2017 - 2 [online]. Website https://www.unodc.org/wdr2017/field/Booklet_2_HEALTH.pdf [accessed 27 03 2018]. and European Drug Report 20172EMCDDA (2017). European Drug Report 2017: Trends and Developments [online]. Website u2987 [accessed 16 12 2019]. heroin is still the leading substance for addiction among high-risk opioids. Heroin addiction is still a widespread problem as a significant cause of morbidity and mortality due to increased risk of cardiopulmonary diseases, chronic infections including Hepatitis C and hepatic failure2. 

The use of surface enhanced Raman spectroscopy (SERS) increases in forensic sciences applications. SERS is a potentially fast and cost-effective analytical method requiring very little amount of sample. There is a large number of SERS based studies dealing with identification of chemical trace evidences and certain body fluids [3–10]. On the other hand, there are few SERS studies regarding identification of toxicological substances including morphine, cocaine and heroin directly dealing with toxicological analysis of heroin and its metabolites with SERS in different biological mediums [11–15]. However, these studies dealt with identification of chemical structure of drugs without measuring their concentrations in biological fluids.

Detection and quantitation of heroin and its metabolites in biological fluids is essential for both healthcare facilities and legal authorities. In healthcare facilities including psychiatric institutions devoted to orehabilitation and treatment of alcohol and drug addiction, enzymatic methods and paper-based tests are used for drug detection, since they are cheaper and easier. However, it is impossible to use these methods for legal purposes because of their false results. In toxicology laboratories, liquid chromatography (LC) coupled with tandem mass spectrometry and gas chromatography mass spectrometry systems are the leading analytical methods to analyze illicit drugs for legal purposes. These quantitative methods are more reliable compared to enzymatic methods, while systems and analyses are quite expensive and time consuming compared to other methods [16,17]. However, SERS is a promising method that allows an accurate identification and quantification of illicit drugs, which can potentially be used, in both healthcare facilities and in toxicology laboratories performing analyses for legal purposes. Therefore, this study based on the question of “Is it possible to measure the concentration of a drug or its metabolites in a biological fluid?”

The aims of this paper are to find out most proper Raman active surface for opiates and accurate SERS spectra of heroin and its metabolites, and develop a potentially more selective and faster method for detection and quantification the concentration of heroin and its metabolites based on SERS measurements in medium of saliva. 

## 2. Materials and methods

### 2.1. Design of study

MM (morphine monohydrate) was used to find out the proper nano-surface for SERS analysis of opiates since morphine has similar chemical structure with many other opiates including heroin and other heroin metabolites. MM as standard material in methanol was diluted with methanol to obtain 250, 500 and 1000 ppm concentrations. These concentrations were used as controls for each other. Diluted MM solutions were mixed with AgNP colloids with 1:1 volume and vortexed. 2 µL of final solutions containing MM concentrations of 125, 250 and 500 ppm were dropped on AgNRs, AuNP/nanocellulose surface, AuNP/polyanilinesurface, TLC silica gel surface and AuNRs. Following an incubation period of 10 minutes, SERS spectra were obtained from at least 5 different area of each sample. Instrument parameters were as follows: 10x objective, 30 μm laser spot size, 150 mW laser power, and 40 s acquisition time. Baseline correction was performed for all measurements. 

### 2.2. Reagents

Methanol for lab analysis, and acetonitrile gradient grade for LC were purchased from Merck KGaA (Darmstadt, Germany). Heroin standard, in powder form, in 99.8% purity. Morphine monohydrate (MM) 1.0 mg/mL calibrated standard solution in methanol, monoacetyl morphine (6MAM) 1.0 mg/mL calibrated standard solution in methanol and morphine-3-β-glucuronide (M3B) 1.0 mg/mL calibrated standard solution in methanol was obtained from Lipomed AG (Arlesheim, Switzerland). Aniline (C6H5NH2) was obtained from Sigma-Aldrich Chemie GmbH (Taufkirchen, Germany) and it was distilled under vacuum before using. Lithium perchlorate (LiClO4) was purchased from Sigma-Aldrich Produktions GmbH (Steinheim, Germany). Nanocellulose, hydroxylamine hydrochloride (NH2OH.HCl), and hydrogen tetrachloroaurate (HAuCl4) were obtained from Sigma-Aldrich Chemie GmbH (Taufkirchen, Germany). Polyethylene glycol (PEG) 400, methanol, silver nitrate (AgNO3), and sodium hydroxide (NaOH) were purchased from Merck KGaA (Darmstadt, Germany). 

### 2.3. Fabrication of silver nano-surface (AgNS) 

AgNS was developed using technique that previously described in the study by Yilmaz et al. [18]. 

### 2.4. Fabrication of AuNPs/nanocellulose surface

The nanocellulose covered AuNPs was prepared by reducing HAuCI4 in the presence of the PEG 400 in an aqueous solution according to the previously reported procedure in the literature [19]. 100 µL of 1 × 10-2 M concentrated aqueous solution of HAuCI4, 0.6 g nanocellulose, 2 mg PEG and 7 mL deionized water were mixed and heated at 90 °C for 20 min to obtain a wine-red solution. The resulting solution was centrifuged, and AuNPs were separated from the unreacted nanocellulose and PEG solution.

### 2.5. The synthesis of AuNPs/polyaniline surface

Reactions were generally carried out in 50 mL vials. 1 mmol of aniline was dissolved in 10 mL of carbon tetrachloride and stirred for a while to obtain a homogenous solution (A) then a certain amount 0.86 g nanocellulose and 1 ml of 1 × 10-2 M concentrated aqueous solution of HAuCI4 were dissolved in 10 mL of 1 M HAuCl4 aqueous solution and after several minutes of stirring, a homogenous solution (B) was obtained. Then solution (A) was transferred into solution (B), to acquire an interfacial reaction between these two phases. After 10 min, green PANI (polyaniline) formed at the interface and then gradually diffused into the aqueous phase. The reaction at the interface was allowed for 2 h. After 2 h, the entire aqueous phase was filled with dark green PANI, while the organic layer was an orange color. Then, the prepared PANI was collected by filtration and washed several times with methanol, acetone, and distilled water. Finally, it was dried in a vacuum oven at 40 °C for 12 h [20]. 

### 2.6. TLC silica gel aluminum sheets

The aluminum side of commercial TLC Silica gel aluminum sheets (Sigma-Aldrich Chemie GmbH, Taufkirchen, Germany) was used to obtain SERS signals of MM in this study.

### 2.7. Surface with gold nanorod arrays (AuNRs)

Anisotropic gold nanorod arrays were produced on the BK7 glass slides via the oblique angle deposition (OAD) technique as previously described in the study by Yilmaz et al.[18]. Due to oblique placement of nanorods the surface had ability to control liquid motion and prevent uncontrolled aggregation.

### 2.8. Silver nanoparticle (AgNP) synthesis

The silver nanoparticles were synthesized by utilizing chemical reduction method suggested by Leopold and Lendl [21]. All chemicals were prepared in double distilled water. In typical experiment, the concentration of silver nitrate was set to 10-3 M in the prepared reaction mixtures. 1.5 × 10-3 M hydroxylamine hydrochloride was used in the final reaction mixture for reduction of silver nitrate. In our experiments, NaOH added to the hydroxylamine solution can adjust the final pH of solution. Solution was mixed vigorously during this process and the color change was distinguishable (pale brown). Finally, this solution was kept for incubation overnight in the dark place.

**Figure 1:**
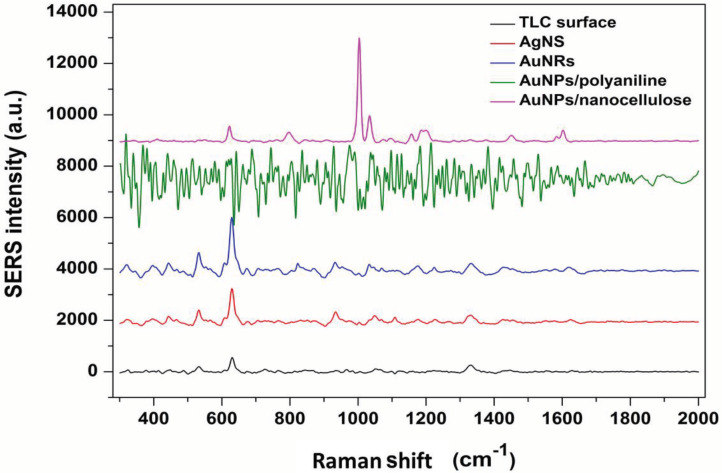
Comparison of SERS spectra of morphine mixed with silver colloid gathered from different surfaces (TLC surface, AgNS, AuNRs, AuNPs/nanocellulose and AuNPs/polyaniline).

After the formation of five different SERS-active substrates (TLC surface, gold nanorods (AuNRs) array, silver nano-surface (AgNS), AuNPs/nanocellulose and AuNPs/polyaniline) for MM detection, MM mixed with silver colloids were dropped on to the substrates, SERS spectra were obtained and given in Figure 1. 

### 2.9. Instrumentation

DeltaNu Examiner Raman microscope (Deltanu Inc., Laramie, WY, USA) with a 785-nm laser source, a motorized microscope stage sample holder, and a CCD detector was used to detect heroin. Instrument parameters were as follows: 10x objective, 30 μm laser spot size, 150 mW laser power, and 40 s acquisition time. Baseline correction was performed for all measurements.

### 2.10. Detection of heroin metabolites concentration using SERS

After the decision of most proper SERS surface, varying concentrations of MM, M3B and 6MAM samples were detected using AuNRs as SERS substrate. The SERS spectra corresponding to each concentration were recorded. The SERS intensity versus concentration calibration curves for MM, M3B and 6MAM (50 ppb–500 ppm) were obtained by calculating the average reading of the response of 5 different areas. The relation between the MM, M3B and 6MAM concentrations and the peak intensity at 627 cm−1 was used for prediction of exponentially. The coefficient of determination (R2) and the detection limit of heroin and its metabolites were calculated. 

### 2.11. SERS procedure and calibration curve for heroin

Heroin in powder form was dissolved in methanol in 5000 ppm concentration. The solution was diluted with methanol to achieve concentrations of 10 ppb, 50 ppb, 1 ppm, 100 ppm, 500 ppm, and 1000 ppm heroin solutions. Equal amount of AgNP solution was added to heroin samples, and final solutions of heroin were in concentrations of 5 ppb, 25 ppb, 500 ppb, 50 ppm, 250 ppm, 500 ppm and 2500 ppm. The relation between the heroin concentrations and the peak intensity at 627 cm−1 was used for prediction of exponentially. The coefficient of determination (R2) and the detection limit of heroin and its metabolites were calculated. 

### 2.12. Biological specimens and heroin and metabolites spikes

Being one of the most common biological fluids analyzed in terms of heroin and its metabolites detection, saliva was used to simulate biological specimens. Saliva samples were obtained from a healthy adult volunteer of free from drug or alcohol use. Stock solution of heroin in methanol in concentration of 1000 ppm was diluted with methanol. 200 µL sample was spiked with heroin, MM, 6MAM and M3B solutions in different concentrations, and vortexed for 5 min. 400 µL of acetonitrile was added to spiked samples to aggregate proteins and to achieve chemical homogenization, and vortexed for 5 min. Solution was centrifuged for 20 minutes in 12,000 rpm. 200 µL of supernatant was taken and mixed with 200 µL AgNP solution and vortexed for 5 min. Solution with AgNP was dropped on to gold surface in amount of 2 µL to gather SERS spectra. Final solution was in concentration of 125 ppm, the similar procedure was performed for the other concentrations (40 ppb, 200 ppb, 1 ppm, 5 ppm and 25 ppm). After an incubation period of 10 min under normal room conditions, SERS spectra were gathered from 5 different points of the spiked sample stains. Mean values of bands’ intensities of 5 spectra were used for each heroin concentration.

### 2.13. Quantification

Spiked heroin, MM, M3B and 6MAM samples in blank saliva matrix were prepared in concentrations of 650 ppb, 30 ppm and 100 ppm to evaluate calibrated formulae and achieve measurements. The author that analyze samples kept blind and did not informed about the concentrations of samples. Spectra of samples were obtained from five different areas and concentrations were evaluated according to intensity-concentration match point in calibration curve for saliva matrix. 

### 2.14. Ethics Approval

Local Ethics Board of Hacettepe University approved this study with decision number 2016/06-02.

## 3. Results

**Figure 2:**
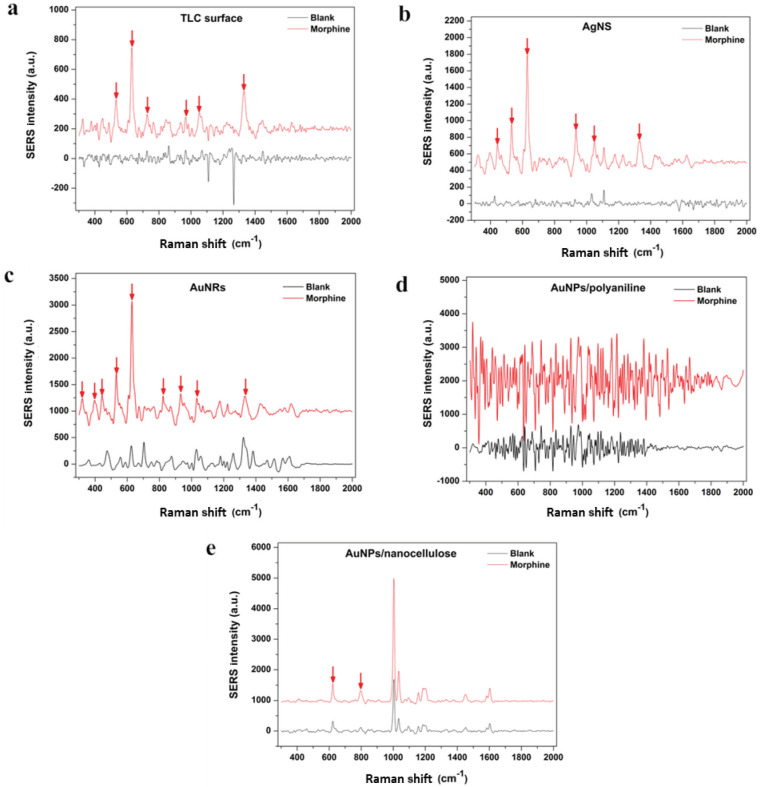
(a) SERS spectra of morphine on and blank surface spectra of TLC surface, (b) AgNS, (c) AuNRs, (d) AuNPs/polyaniline, (e) AuNPs/nanocellulose (Red arrows show strong bands of morphine spectra).

The SERS spectra of each developed SERS-substrates were examined according to blank and MM samples (Figure 2). After statistically determining extreme values as strong bands and excluding bands associated with surface itself, TLC surface showed six strong bands at 532, 627, 726, 967, 1049 and 1332 cm-1 (Figure 2a). There were also seven strong bands gathered MM spectra from AgNS surface at 443, 532, 627, 934, 1049, 1109 and 1332 cm-1, respectively (Figure 2b). AuNRs nano-surface produced the most intense bands at eight different SERS bands at 321, 443, 534, 627, 823, 935, 1033 and 1332 cm-1(Figure 2c). AuNPs/polyanniline surface revealed the most intensive noise amongst all five surfaces. There was no identifiable band exceeding the noise level (Figure 2d). AuNPs/nanocellulose surface showed the least back noise (Figure 2e). However, it revealed only two strong bands at 627 and 797 cm-1after exclusion of bands assigned to surface.

**Figure 3:**
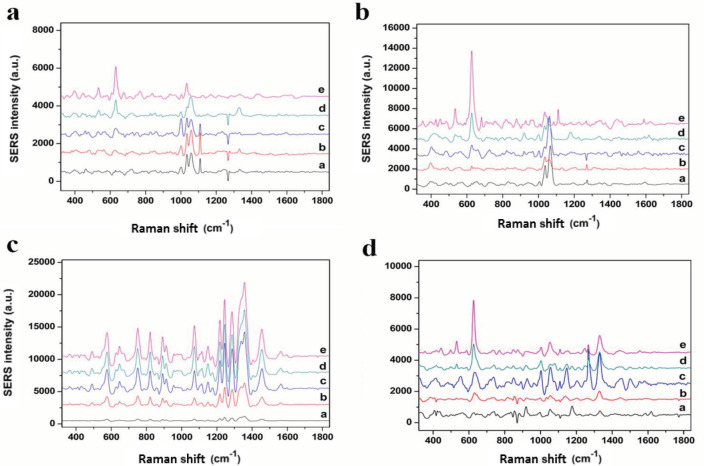
C-C_alicyclic,aliphatic_ vibration bands at 627 cm^-1^ of (a) MM, (b) M3B, (c) 6MAM, (d) Heroin from 20 ppb to 125 ppm concentration in saliva (a) 20 ppb (b) 1 ppm, (c) 5 ppm, (d) 25 ppm, (e) 125 ppm.

After determining the most proper surface for opiates by evaluating SERS signals of MM, AuNRs were used in detection and quantification of heroin and its metabolites. The spectra of heroin, M3B and 6MAM obtained using SERS is shown in Figure 3. 

**Figure 4:**
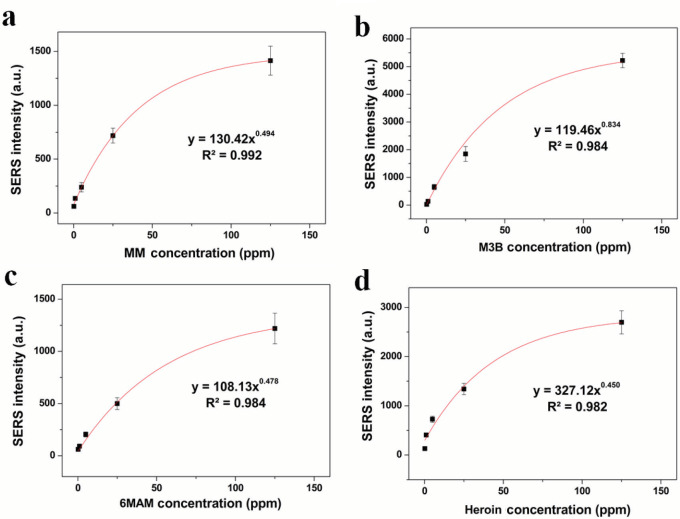
Calibration curve for (a) MM, (b) M3B, (c) 6MAM, (d) Heroin samples in saliva at a concentration range of 200 ppb–125 ppm obtained by using AuNRs and Ag colloids.

Heroin and its metabolites spectra have one prominent and consistent band at 627 cm-1 and this could be identified in extremely low concentrations. Heroin spectra also showed strong bands at 443, 531, 1107, 1245 and 1338 cm-1. Being the most stable band, intensities of the band at 627 cm-1 were utilized for calibration curves for heroin and its metabolites (Figure 4). 

The analyses of parameters for MM, M3A, 6MAM and heroin samples using AuNRs as SERS substrate were evaluated in Table 1. 

**Table 1:** Evaluation of the analysis parameters of MM, M3B, 6MAM and heroin.

	MM*	M3B**	6MAM***	Heroin
Equation	y=130.42x0.494	y=119.46x0.834	y=108.13x0.478	y=327.12x0.450
R2 value	0.992	0.984	0.984	0.982
LOD (ppb)	20.91	17.01	19.91	31.69
LOQ (ppb)	62.74	51.03	59.73	95.06

* MM: Morphine monohydrate, ** 6MAM: 6-Monoacetyl morphine, *** M3B: Morphine-3-β-glucuronide.

Since the saliva samples had the most consistent and stable bands with higher intensities, saliva matrix used in quantification studies. Gathered spectra of five different concentrations in saliva were used to create calibration curves for heroin, MM, M3B, and 6MAM-spiked saliva. 

To measure heroin and its metabolites concentration in saliva, blind samples of spiked saliva were prepared by one of the authors, and a blinded author quantified heroin concentration in three samples. Spectra of blind samples were gathered from five different area of the staining and mean values of the intensities of the bands at 627 cm-1 used in calculation. Concentrations were calculated using intensity-concentration match point in calibration curve. For this reason, calculations were made according to equation of “CH = (I/327.12)1/0,45” for heroin, “CMM = (I/130.42)1/0,494” for MM, “CM3B = (I/119.46)1/0,834” for M3B and “C6MAM = (I/108.13)1/0,478” for 6MAM (C: concentration, I: intensity).

Spiked concentrations of heroin, MM, M3B and 6MAM in saliva matrix and calculated concentrations are presented in Table 2. 

**Table 2:** Known concentrations of spiked saliva sample, mean value of the intensity of the band at 627 s^-1^ and calculated concentrations of blind samples.

Sample	Known concentration (ppb)	Calculated concentration (ppb)
MM*	650	620.46±28.12
	30000	31250.9±1989.48
	100000	98356.4±1214.56
M3B**	650	673.35±36.44
	30000	29878.4±485.87
	100000	98503±781.94
6MAM***	650	634.92±18.2
	30000	29002.5±665.42
	100000	101541±1779.4
Heroin	650	632.71±21.71
	30000	28844.24±1298.83
	100000	98819.35±649.8

* MM: Morphine monohydrate, ** M3B: Morphine-3-β-glucuronide, *** 6MAM: 6-Monoacetyl morphine.

Total duration for analysis of heroin concentration in a heroin-spiked saliva sample using SERS was approximately 20 min. This is much lower compared to conventional instrumental analyses procedure in daily routine of toxicology labs. 

## 4. Discussion

Abuse of illicit drugs or narcotic medications has been a challenging issue for both healthcare and legal systems for several centuries [2]. Therefore, identification and/or quantification of illicit drugs or certain medications is crucial for healthcare and forensic professionals. For this purpose, there are several methods investigated in the literature, however there is still need for cheaper and faster methods for toxicological analyses [11,12,22–24]. 

SERS spectra of MM obtained in current study were compatible with Raman and SERS spectra revealed in the previously conducted studies [24–26]. Li et al. [26] used Ag film as a Raman-active material and gathered SERS spectra of MM using 633-nm laser source. They showed the most intense bands at 566, 633, 850, 1123, 1205 and 1284 Raman wavenumbers. Rana et al. [25] studied SERS spectra of MM using Ag colloids with both 785- and 633-nm laser sources. They stated that characteristic MM’s Raman bands detected by 633-nm laser source were more remarkable, which were seen at 462, 542, 609, 625, 1350, 1469 and 1698 cm-1. The results in the present study were compatible with the previous studies and showed the most intense and characteristic bands at 321, 443, 534, 627, 823, 935, 1033 and 1332 cm-1.

Ag nanoparticles are known to intensify Raman signals of MM as well as AuNRs [11,12]. Inscore et al. [12] studied 80 illicit drugs and their metabolites using SERS and stated that best results can be achieved with AuNRs to reveal SERS spectra of MM. In addition to the literature, we achieved to increase reliability of analysis by avoiding uncontrolled aggregation of studied sample due to a surface characterized with angled AuNRs that potentially control liquid motion. A comparison of Raman-active quality of studied surfaces in terms of SERS findings and related comments is shown in Table 3.

**Table 3:** Comparison of Raman-active quality of studied surfaces in terms of SERS findings.

Surface	SERS findings and related comment
AgNS	· Second least background noise · Second most efficient results · More identifiable peaks with higher intensities compared AuNP/Nanocellulose, TLC and aniline
AuNRs	· Third least background noise · The most efficient results · More identifiable peaks in higher intensities compared to all
AuNPs/nanocellulose	· The least background noise · The intensity of main band (627 s-1) was significantly lower compared to AgNRs and AuNRs
TLC	· Lower number of identifiable bands with extremely low intensities · Useless in SERS-based detection and quantification of MM*
AuNPs/polyaniline	· The most background noise.· The least number of identifiable peaks · Useless in SERS-based detection and quantification of MM*.

* MM: Morphine monohydrate.

In the literature, there are few previous studies dealing with Raman spectra of heroin and its metabolites as an illicit drug. Hodges et al. [27] studied Raman spectra of certain drugs of abuse including cocaine and heroin, and they have shown the most prominent band between 620–630 cm-1for heroin, in their study. A similar band formation has been shown in Ryder et al.’s study [28], which had also showed Raman spectra of heroin with more identifiable bands gathered from solid mixtures. However, these studies had not revealed an exact Raman spectrum for heroin. Since it is more sensitive and specific compared to conventional Raman spectroscopy, SERS analyses would reveal more convenient and useful data.

To the best of our knowledge, this is the first ever study dealing with SERS spectra of heroin and its metabolites measurement in saliva medium. The surface produced according to the method by Yilmaz et al. [18] and AgNP were utilized to reveal better intensities in extremely low heroin concentration in order to use it for identification and quantification of heroin in complex media. In our study, band at 627 cm-1 was the most prominent and convenient band of which intensity was also identifiable in low concentrations for all four analytes.

SERS is a fast and accurate way to identify and quantitate illicit drugs. Handheld spectroscopy also has potential to provide opportunity to analyze any samples in the field in a fast and accurate way Ocean Optics (2017). Identifying Illicit Drugs using Surface Enhanced Raman Spectroscopy [online]. Website http://blog.oceanoptics.com/illicit-drugs-identification-sers [accessed 04 09 2018]. [23,29]. Although there are many commercial handheld Raman systems being sold for detection of illicit drugs in the market, most of these systems cannot overcome fluorescence effectively and have relatively lower signal/noise ratios [25,29–31]. SERS aids in overcoming fluorescence and increasing signal/noise ratios [10–12,25]. Therefore, SERS needs to be used to identify and measure heroin concentrations in complex media such as body fluids namely saliva, urine, and blood. 

Inscore et al. studied identification of certain illicit drugs in saliva with SERS including heroin without measuring their concentrations. They have stated that heroin is Raman active on gold nano-surface [11,12]. Andreou et al. [29] also studied detection of illicit drugs in saliva with a micro-fluidic device using SERS and AgNPs. In this study, we have proposed a SERS method to detect and quantify heroin with a specific gold nano-surface produced with gold nano-arrays in a specific position to control liquid motion [18] and AgNP as Raman signal intensifiers. SERS spectra obtained with our method showed significantly higher intensities and low signal/noise ratios in bands specific to heroin. 

A study from China [22] revealed that drugs could be identified in urine samples via SERS-based systems. A recent study [13] showed that morphine identification is possible in human urine with SERS. Similarly, our results supported the information that identification of heroin and morphine is possible in saliva samples. 

Currently utilized quantitative analytical toxicology procedures are more reliable compared to enzymatic methods, while systems and analyses are quite expensive and time consuming. A routine analysis of heroin concentration in a biological fluid with LC–MS or GS–MS took 60 to 90 minutes, while the presented SERS procedure took approximately 20 min [32]. Therefore, the present study suggests a potentially cheaper and faster method than routine heroin analysis systems for heroin analysis in biological fluids. As a major limitation of the study, the utilized method might not be able to provide similar results in patients with certain diseases affecting the quality and contents in biological fluids, namely saliva. 

In conclusion, obtained results showed that heroin, MM, M3B and 6MAM can be detected and quantified in saliva samples using a SERS-based system. Additionally, the present study revealed that synergetic effect of a specific gold nano-surface with ability controlling liquid motion and silver nanoparticles increase band numbers and intensities. Therefore, we suggest a fast, accurate and cost-effective method to detect and quantify heroin in biological fluids, saliva in particular.

## Acknowledgments

This work was supported financially by Hacettepe University (Grant Number: THD-2018-17241). This article is based on studies of PhD thesis of Ramazan AKÇAN in Bioengineering Department of Graduate School of Science and Engineering. This study was presented in Taiwan-Turkey Science Summit that was held in Ankara, Turkey, on 1-4 April 2018.  Authors would to acknowledge Assoc. Prof. Gökhan DEMİREL for his technical support and material supply support.

## Informed consent

There is no need informed consent about this work.

This study was presented at the Taiwan-Turkey Science Summit entitled “Translation of Cells, Nanomaterials and Signaling Molecules into Regenerative Medicine” between April 1 to 3, 2018.
